# Epigenomic translocation of H3K4me3 broad domains over oncogenes following hijacking of super-enhancers

**DOI:** 10.1101/gr.276042.121

**Published:** 2022-07

**Authors:** Aneta Mikulasova, Daniel Kent, Marco Trevisan-Herraz, Nefeli Karataraki, Kent T.M. Fung, Cody Ashby, Agata Cieslak, Shmuel Yaccoby, Frits van Rhee, Maurizio Zangari, Sharmilan Thanendrarajan, Carolina Schinke, Gareth J. Morgan, Vahid Asnafi, Salvatore Spicuglia, Chris A. Brackley, Anne E. Corcoran, Sophie Hambleton, Brian A. Walker, Daniel Rico, Lisa J. Russell

**Affiliations:** 1 Biosciences Institute, Newcastle University, Newcastle upon Tyne NE2 4HH, United Kingdom;; 2 Translational and Clinical Research Institute, Newcastle University, Newcastle upon Tyne NE2 4HH, United Kingdom;; 3 Department of Biomedical Informatics, University of Arkansas for Medical Sciences, Little Rock, Arkansas 72205, USA;; 4 Université de Paris (Descartes), Institut Necker-Enfants Malades (INEM), Institut National de la Santé et de la Recherche Médicale (Inserm) U1151, and Laboratory of Onco-Hematology, Assistance Publique-Hôpitaux de Paris, Hôpital Necker Enfants-Malades, 75015 Paris, France;; 5 Myeloma Center, University of Arkansas for Medical Sciences, Little Rock, Arkansas 72205, USA;; 6 NYU Langone Medical Center, Perlmutter Cancer Center, NYU Langone Health, New York, New York 10016, USA;; 7 Aix-Marseille University, Inserm, Theories and Approaches of Genomic Complexity (TAGC), UMR1090, 13288 Marseille, France;; 8 Equipe Labellisée Ligue Contre le Cancer, 13288 Marseille, France;; 9 SUPA, School of Physics and Astronomy, University of Edinburgh, Edinburgh EH9 3FD, United Kingdom;; 10 Lymphocyte Signalling and Development Programme, Babraham Institute, Cambridge CB22 3AT, United Kingdom;; 11 Great North Children's Hospital, Newcastle upon Tyne Hospitals NHS Foundation Trust, Newcastle upon Tyne NE1 4LP, United Kingdom;; 12 Melvin and Bren Simon Comprehensive Cancer Center, Division of Hematology Oncology, Indiana University, Indianapolis, Indiana 46202, USA

## Abstract

Chromosomal translocations are important drivers of haematological malignancies whereby proto-oncogenes are activated by juxtaposition with enhancers, often called *enhancer hijacking*. We analyzed the epigenomic consequences of rearrangements between the super-enhancers of the immunoglobulin heavy locus (*IGH*) and proto-oncogene *CCND1* that are common in B cell malignancies. By integrating BLUEPRINT epigenomic data with DNA breakpoint detection, we characterized the normal chromatin landscape of the human *IGH* locus and its dynamics after pathological genomic rearrangement. We detected an H3K4me3 broad domain (BD) within the *IGH* locus of healthy B cells that was absent in samples with *IGH-CCND1* translocations. The appearance of H3K4me3-BD over *CCND1* in the latter was associated with overexpression and extensive chromatin accessibility of its gene body. We observed similar cancer-specific H3K4me3-BDs associated with hijacking of super-enhancers of other common oncogenes in B cell (*MAF*, *MYC*, and *FGFR3/NSD2*) and T cell malignancies (*LMO2*, *TLX3*, and *TAL1*). Our analysis suggests that H3K4me3-BDs can be created by super-enhancers and supports the new concept of *epigenomic translocation*, in which the relocation of H3K4me3-BDs from cell identity genes to oncogenes accompanies the translocation of super-enhancers.

In healthy cells, many proto-oncogenes and tumor suppressor genes coordinate to control cell proliferation. However, these essential genes can be activated or inactivated by different genomic alterations to their coding sequence, including nucleotide substitutions, gene amplification or loss, and gene fusion. Proto-oncogenes can also be converted to oncogenes without alterations to the protein-coding sequence; structural alterations can result in juxtaposition of proto-oncogenes and super-enhancers promoting their overexpression, a situation that is referred to as *enhancer adoption* (Lettice et al. 2011) or, more often, *enhancer hijacking* (Northcott et al. 2014; Beroukhim et al. 2016; Weischenfeldt et al. 2017). Other structural alterations that disrupt local chromatin architecture can produce epigenomic activation of proto-oncogenes to resemble adjacent chromosomal neighborhoods (Hnisz et al. 2016). However, little is known about the epigenomic consequences of enhancer hijacking on the deregulated oncogenes. To begin to understand how structural alterations activate proto-oncogenes, we have analyzed the changes in chromatin states associated with super-enhancer translocation events in cancer.

Trimethylation of lysine 4 on histone H3 (H3K4me3) is a chromatin modification classically associated with the promoters of transcriptionally active genes (Howe et al. 2017) and also is present at some active enhancers (Pekowska et al. 2011; Shen et al. 2016; Hu et al. 2017; Russ et al. 2017; Li et al. 2019). Although H3K4me3 marks tend to show sharp 1- to 2-kb peaks around promoters, some genes have broader regions of H3K4me3, also known as H3K4me3 broad domains (H3K4me3-BDs) that expand over part or all of the coding sequence of the gene (up to 20 kb) (Pekowska et al. 2010, 2011; Benayoun et al. 2014; Chen et al. 2015). These H3K4me3-BDs are associated with cell-identity genes (Pekowska et al. 2010; Benayoun et al. 2014) and cell-specific tumor suppressor genes (Chen et al. 2015), where they favor transcriptional consistency and increased expression (Benayoun et al. 2014). It has been observed that some tumor suppressor genes show a narrower breadth of these domains in cancer, associated with their down-regulation in malignant cells (Chen et al. 2015).

Super-enhancers were originally defined as enhancers with unusually high levels of certain transcriptional coactivators (Lovén et al. 2013; Whyte et al. 2013) and a median size >8 kb (Pott and Lieb 2015). Recent studies of 3D genome networks suggest that the presence of H3K4me3-BDs in genes is associated with increased interactions with active super-enhancers (Cao et al. 2017; Thibodeau et al. 2017). More recently, Dhar and coworkers (2018) have shown that the histone methyl-transferase MLL4 is essential for the methylation of both super-enhancers and H3K4me3-BDs and proposed a hypothetical model whereby MLL4 would be essential to maintain the interaction of super-enhancers and tumor suppressor genes with BDs. However, the data from these previous studies could not address whether H3K4me3-BDs are formed as a consequence of super-enhancer activity.

The immunoglobulin (Ig) loci contain powerful super-enhancers to drive antibody formation and expression. The immunoglobulin heavy (*IGH*) locus has four regions encompassing: constant (C_H_), joining (J_H_), diversity (D_H_), and variable (V_H_) gene segments. The human C_H_ region encodes nine different Ig isotypes: *IGHA2*, *IGHE*, *IGHG4*, *IGHG2*, *IGHA1*, *IGHG1*, *IGHG3*, *IGHD*, and *IGHM* (Supplemental Fig. S1). Complex regulatory and genomic rearrangements in the *IGH* locus are required to ensure that Ig transcripts containing only one of each of these gene segments are expressed in each B cell at the correct stage of B cell differentiation. These natural processes predispose human Ig loci to inappropriate translocation events. As a result, these super-enhancer-rich regions are commonly hijacked in B cell malignancies, providing the optimal model in which to address whether H3K4me3-BDs are formed as a consequence of super-enhancer activity.

## Results

### Identification of H3K4me3-BDs using the chromatin state model

H3K4me3-BDs have been defined in different ways, using size- and height-ranked (Benayoun et al. 2014; Chen et al. 2015, respectively) H3K4me3 peaks and, more recently, using a selection method based on two (intermediate and high) inflection points of ranked H3K4me3 peaks (Belhocine et al. 2022). However, all identified the enrichment of similar biological processes related to cell identity. In this study, we defined H3K4me3-BDs and super-enhancers using the chromatin state model developed by Carrillo-de-Santa-Pau et al. (2017) using BLUEPRINT data of H3K4me3, H3K4me1, H3K27ac, H3K27me3, H3K9me3, and H3K36me3 (Supplemental Tables S1–S3). We defined H3K4me3-BDs as domains with H3K4me3 >2 kb and compared them across different hematopoietic cell types (Supplemental Results; Supplemental Table S4; Supplemental Fig. S2). We observed shared H3K4me3-BDs in all of the cell types that were associated with basic cellular functions (Supplemental Fig. S3D). Only a small proportion of detected H3K4me3-BDs were cell type–exclusive (Supplemental Fig. S2), identifying cell-identity genes (Supplemental Fig. S3A–C) as reported by the other studies mentioned above. With recent studies observing increased interactions between H3K4me3-BD and super-enhancers (Cao et al. 2017; Thibodeau et al. 2017), we also assessed the proximity of super-enhancers (defined as domains with H3K4me1 and H3K27ac >5 kb) to genes marked with narrow promoter-restricted H3K4me3 peaks or H3K4me3-BDs. In agreement with previous reports, we identified a significantly higher proportion of H3K4me3-BD having a proximal super-enhancer(s) within 100 kb in comparison to genes marked with narrow promoter-restricted H3K4me3 peaks (Supplemental Fig. S4). The cell type–exclusive H3K4me3-BDs (Supplemental Fig. S4D–F) had proximal super-enhancer(s) more frequently than cell type–nonexclusive H3K4me3-BDs (Supplemental Fig. S4A–C).

### Cancer-associated translocation events provide opportunities to investigate super-enhancer and H3K4me3-BD relationships

A common translocation in B cell malignancies involves the proto-oncogene cyclin D1 (*CCND1*) at 11q13 and *IGH* at 14q32 (*IGH-CCND1*). To begin to understand how this translocation event leads to *CCND1* activation, we characterized the epigenomic landscape in these two regions from healthy and malignant B cells. To precisely define the location and activity of the human *IGH* super-enhancers and promoters and the chromatin dynamics of the *CCND1* locus in B cells, we used 108 chromatin state maps for different hematopoietic cell types built with over 700 ChIP-seq data sets from BLUEPRINT (Supplemental Tables S1–S3; Stunnenberg et al. 2016; Carrillo-de-Santa-Pau et al. 2017). These data included samples from hematopoietic stem cells, four stages of healthy B cell differentiation, plasma cells and both primary tumor cells, and cell lines derived from four different B cell hematological malignancies. We used seven healthy non–B cell cell types (T cells, neutrophils, eosinophils, monocytes, macrophages, erythroblasts, and megakaryocytes) as negative controls in which chromatin activity signals in the *IGH* locus are not predicted.

### Epigenomic cartography of the human *IGH* locus

Three super-enhancers have been previously described in the nonvariable region of *IGH*: Eα2, Eα1, and Eμ (Mills et al. 1983, 1997; Chen and Birshtein 1997). However, the precise definition, location, and activity of these regulatory regions throughout the different stages of healthy human B cell differentiation and human B cell–derived malignancies have not been previously investigated.

**Figure 1.**
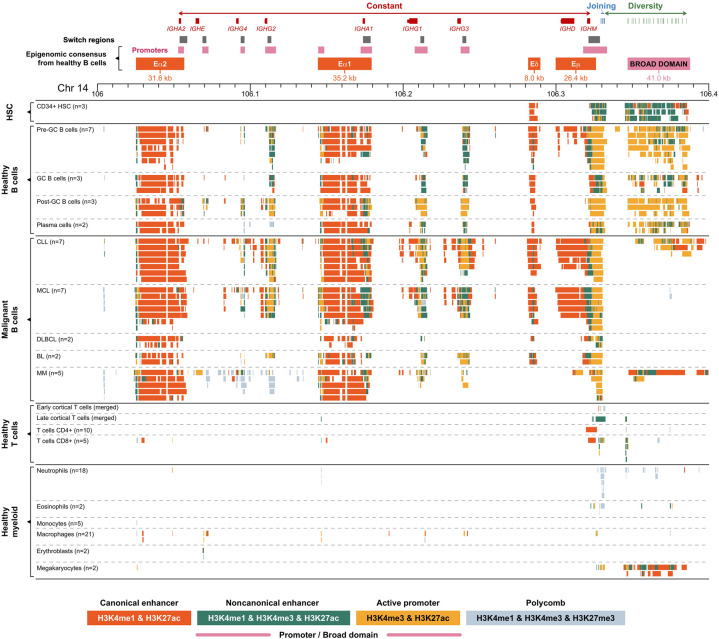
Genomic and epigenomic architecture of the *IGH* locus (14q32) in healthy and malignant human hematopoietic cells. Each panel represents collapsed cell type–specific signal of ChIP-seq chromatin states included in this study (Supplemental Table S1). AID motif clusters were detected as high enrichment of AID motifs (more than 200 of RGYW motifs per 2.5 kb). (HSCs) Hematopoietic stem cells, (GC) germinal center, (CLL) chronic lymphocytic leukemia, (MCL) mantle cell lymphoma, (DLBCL) diffuse large B cell lymphoma, (BL) Burkitt lymphoma, and (MM) multiple myeloma.

The three known B cell–specific active enhancers (high signal of both histone marks H3K4me1 and H3K27ac; ∼40 kb) were found within C_H_ region at the following genomic locations: Chr 14: 106,025,200–106,056,800 (Eα2), Chr 14: 106,144,200–106,179,400 (Eα1), and Chr 14: 106,281,800–106,326,200 (Eμ). The Eμ enhancer showed dynamic changes associated with the B cell development stage. A larger region appears active in naive B cells before entering the germinal center (GC), whereas in post-GC B cells, the region of activity is reduced (class-switched memory B cells and plasma cells) (Fig. 1). A similar area of reduced activity was also observed in malignant samples derived from cells after class-switch recombination, including diffuse large B cell lymphoma (DLBCL), Burkitt lymphoma (BL), and multiple myeloma (MM) in comparison to pre-GC-like malignancies such as mantle cell lymphoma (MCL). We propose that a second, independent enhancer should be distinguished from Eµ based on the observation of a ∼10-kb gap between these two enhancer regions that is supported by high-quality mapping. We refer to this as Eδ (Eδ at Chr 14: 106,281,800–106,289,800 and Eμ at Chr 14: 106,299,800–106,326,200) (Fig. 1). None of these four enhancer regions were detected in healthy myeloid and T cells.

### Epigenomic cartography of the human *CCND1* gene

**Figure 2.**
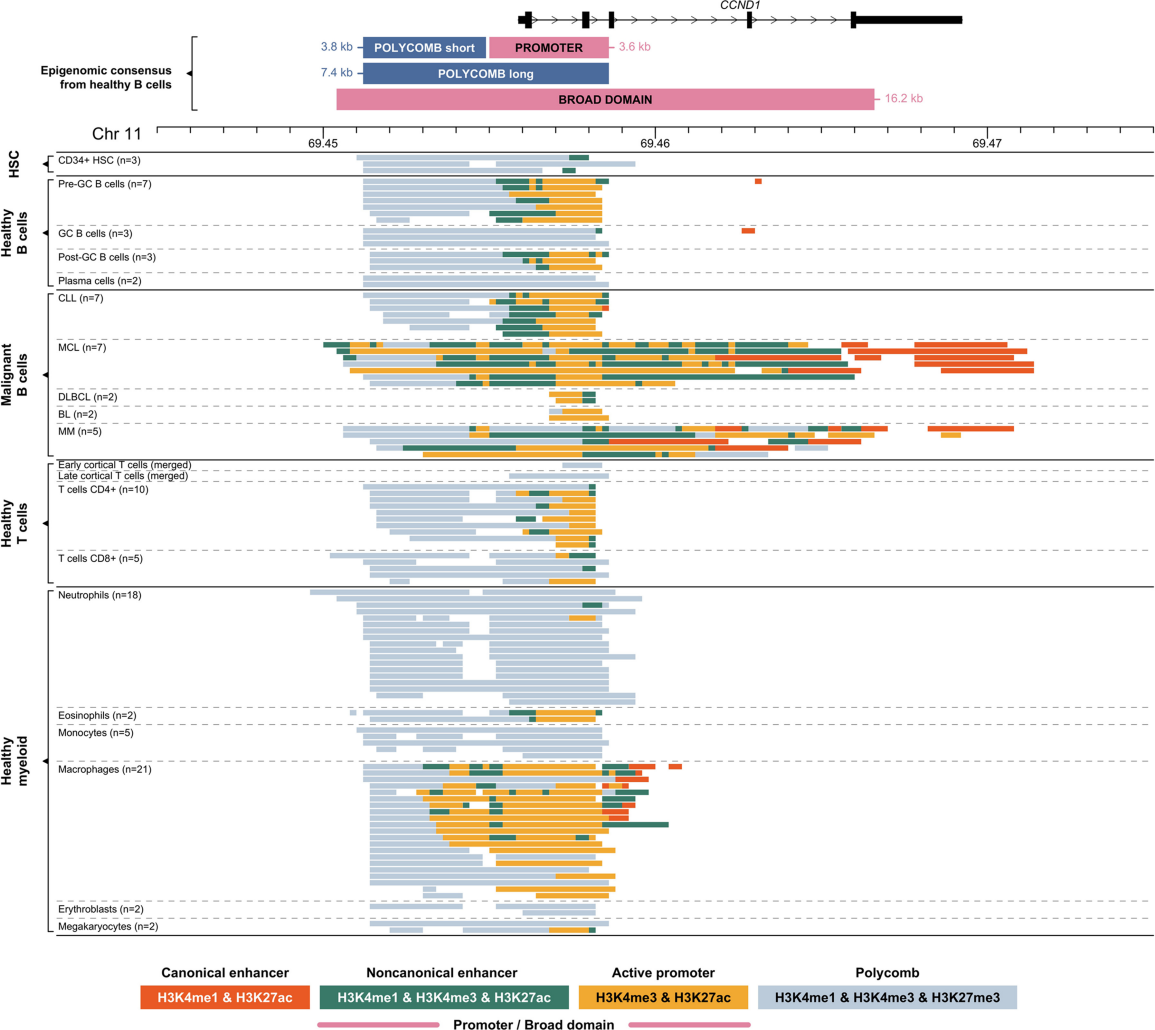
Genomic and epigenomic architecture of the *CCND1* locus (11p13) in healthy and malignant human hematopoietic cells. Each panel represents collapsed cell type–specific signal of ChIP-seq chromatin states included in this study (Supplemental Table S1). Abbreviations are the same as given in the legend of Figure 1.

The proto-oncogene *CCND1* is one of the most common *IGH* translocation partner genes in hematological malignancies, a hallmark of MCL (Vose 2017) and frequently observed in MM (Walker et al. 2013). The chromatin states in the *CCND1* promoter and immediate upstream region (Fig. 2) suggest that the Polycomb repressive complex may be implicated in the regulation of this cell cycle gene in healthy human B cells. When the promoter is inactive in nonproliferating GC B cells and terminally differentiated plasma cells, the Polycomb state covers the whole promoter and upstream region (Polycomb long) (Fig. 2). The Polycomb state is maintained upstream of the promoter in healthy proliferating hematopoietic cell types, including pre-GC and post-GC B cells, some T cells, macrophages, and eosinophils (Polycomb short) (Fig. 2).

We observed a different chromatin landscape for *CCND1* across four hematological malignancy subtypes (Fig. 2). In the majority of cases, the *CCND1* promoter shows an active state. Chronic lymphocytic leukemia (CLL) samples show a similar pattern to healthy B cells, with the Polycomb state domain upstream of the active promoter. DLBCL and BL samples show a narrow H3K4me3 active promoter but lack the upstream Polycomb domain. All MCL and some MM samples have a larger active promoter/enhancer region extending upstream of and downstream from the transcription start site, with most of the gene body containing H3K4me3 marks, a pattern that looks comparable to an H3K4me3-BD (Pekowska et al. 2010, 2011; Benayoun et al. 2014; Chen et al. 2015). Cytogenetic analysis for BLUEPRINT primary patient samples confirmed the presence of an *IGH-CCND1* rearrangement in all MCL patient samples. Although we were unable to confirm this for the MM samples, it is reported that 20% of MM patients will have an *IGH-CCND1* rearrangement (Avet-Loiseau et al. 2007). We therefore hypothesized that the juxtaposition of an *IGH* super-enhancer close to the coding region of *CCND1* results in the presence of an H3K4me3-BD over the coding region of the gene.

### Relocation of an H3K4me3-BD from *IGH* to *CCND1* as a consequence of a hijacked super-enhancer

We discovered a B cell–specific H3K4me3-BD at the genomic location, Chr 14: 106,346,800–106,387,800 (41 kb), overlapping the D_H_ region of the *IGH* locus (Fig. 1). This element was characterized by high signal of both H3K4me3 and H3K27ac (typical for promoters), combined with occasional H3K4me1 high signal. This H3K4me3-BD is absent or significantly reduced in malignant B cells. These data suggest that the disappearance of the H3K4me3-BD from the *IGH* locus may be the consequence of *IGH* super-enhancer hijacking via genomic translocation events that are known to be present in the malignancy subtypes represented (Supplemental Tables S2, S3).

**Figure 3.**
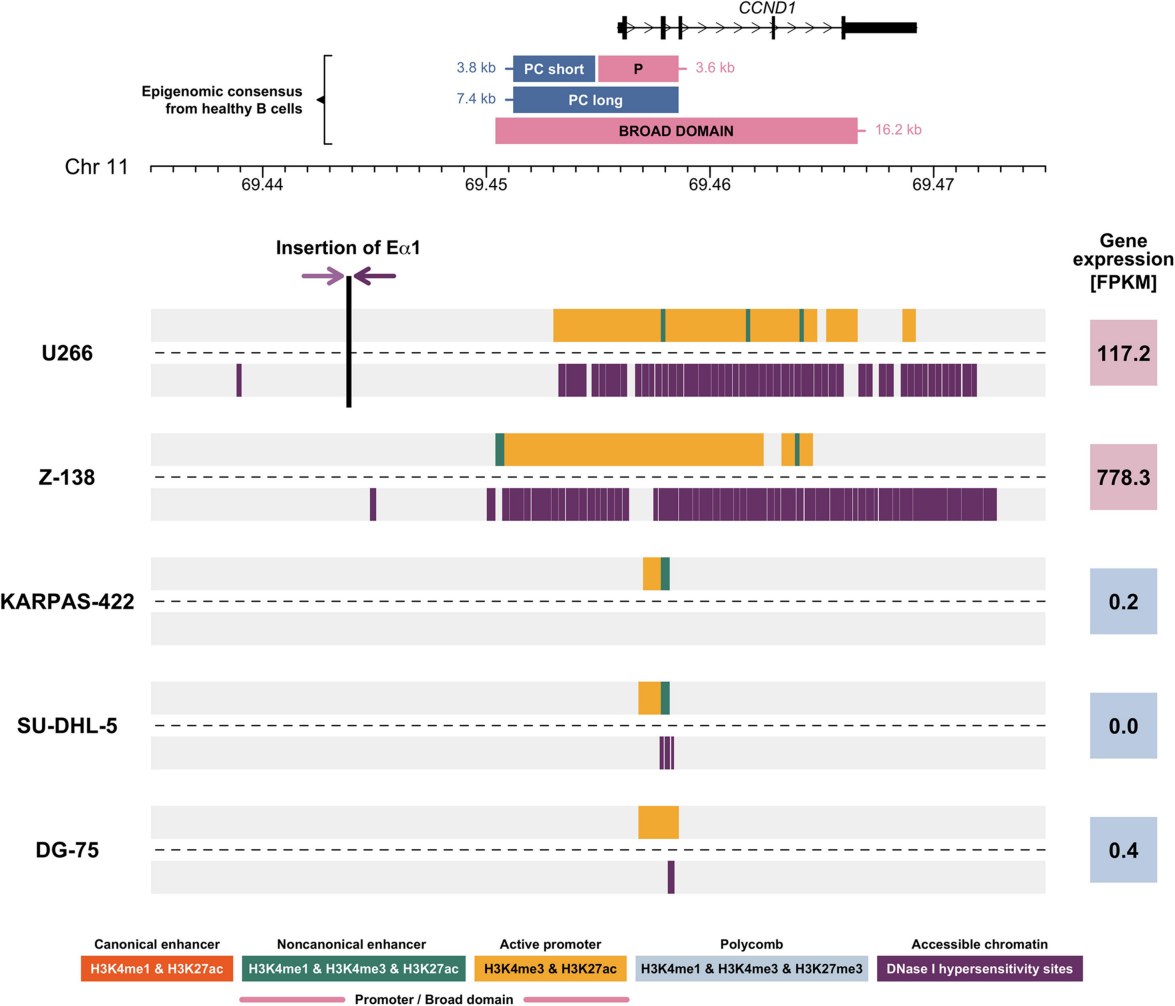
Chromatin landscape of the *CCND1* locus in five cell lines derived from B cell hematological malignancies. The *upper* line of each cell line (Supplemental Table S3) represents the selected ChIP-seq chromatin states (Supplemental Table S1), and the *lower* line shows DNase I hypersensitivity sites for the nonvariable region of the *IGH* locus. The vertical black line in U266 marks position of the inserted Eα1 super-enhancer. The breakpoints within the *IGH* locus are characterized in Supplemental Figure S5. Shaded-purple arrows symbolize translocation orientation. Numbers in colored squares (red denotes high expression; blue, low expression) show *CCND1* expression detected using RNA-seq in fragments per kilobase of transcript per million mapped reads (FPKM). (PC) Polycomb, (P) promoter.

To further investigate the presence or absence of H3K4me3-BD as a result of *IGH* translocations, we studied the MM cell line U266, in which the *IGH* super-enhancer Eα1 is inserted next to the *CCND1* proto-oncogene (Gabrea et al. 1999). This cell line provides the opportunity to analyze the consequence of the relocation of an isolated *IGH* super-enhancer. Using paired-end read targeted DNA sequencing, we precisely mapped the chromosomal changes at the *IGH* locus (Supplemental Fig. S5). We confirmed two chromosomal breakpoints that occur in the *IGHE* and *IGHA* switch regions and result in the cut and paste of the *IGH* Eα1 super-enhancer into Chromosome 11, ∼12 kb upstream of *CCND1* (Fig. 3). The vast majority of the H3K4me3-BD observed over the D_H_ region in healthy B cells is absent from this translocated cell line. A small region of H3K4me3 is still observed over the D_H_ region, perhaps owing to the presence of the *IGH* Eα2 super-enhancer that enables the expression of the IgE monoclonal Ig produced by U266 cells (Hellman et al. 1988). Importantly, a cancer-specific H3K4me3-BD is observed covering most of the *CCND1* gene body (Fig. 3). This observation suggests the presence of an *epigenomic translocation* of the H3K4me3-BD from *IGH* to the *CCND1* locus, present exclusively in MCL and MM samples (Figs. 1, 2). This epigenomic translocation can result from both errors in VDJ recombination (Stamatopoulos et al. 1999), as observed in MCL, and class-switch recombination (Walker et al. 2013), as observed in MM. An aberrant H3K4me3-BD over the adjacent gene *MYEOV* was also observed in the U266 cell line (Supplemental Fig. S6). This suggests that the inserted Eα1 super-enhancer has a bidirectional effect, generating an H3K4me3-BD over another adjacent gene.

### Relocation of H3K4me3-BD is associated with increased chromatin accessibility and transcription

To understand the effect of the epigenomic translocation on chromatin accessibility, we used BLUEPRINT DNase I hypersensitivity data available for five cell lines derived from B cell malignancies (Supplemental Table S3). Two of the cell lines had an *IGH-CCND1* rearrangement, including U266 (already described above) and the MCL cell line Z-138. We observed the H3K4me3-BD and strong DNase I hypersensitivity signals encompassing the entire coding region of the *CCND1* gene in both cell lines (Fig. 3). The increased chromatin accessibility extended beyond the coding region of *CCND1*. This pattern is associated with an increase in *CCND1* expression in U266 and Z-138 (Fig. 3). The presence of the H3K4me3-BD, the strong DNase I hypersensitivity signal, and increased transcript levels for *CCND1* were not observed in the remaining three cell lines without the *IGH-CCND1* rearrangement (Fig. 3). Although *MYEOV* showed an H3K4me3-BD, increased chromatin accessibility, and expression in U266, this was not observed in Z-138, whereby the reciprocal translocation results in *MYEOV* being retained on the derived Chromosome 11, with the relocation of *CCND1* to Chromosome 14 juxtaposing it to all three *IGH* super-enhancers (Guikema et al. 2005; Rico et al. 2022).

### Cancer-specific H3K4me3-BDs are associated with Ig translocations in MM

**Figure 4.**
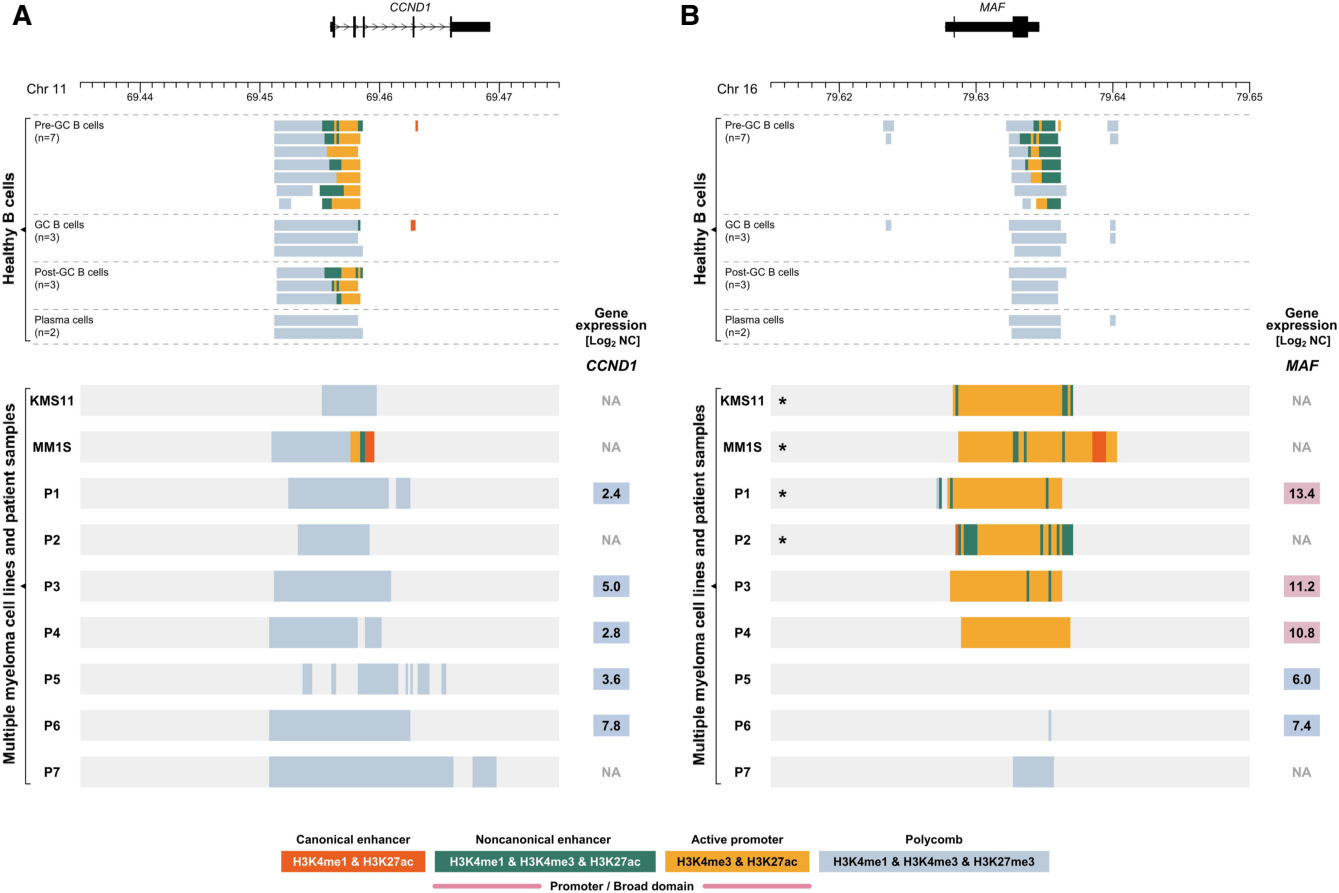
Chromatin landscape of the *CCND1* (11q13) and *MAF* (16q23) loci in healthy human B cells and multiple myeloma (MM) samples. *Upper* panels show selected ChIP-seq chromatin states (Supplemental Table S1) of *CCND1* (*A*) and *MAF* (*B*) loci in BLUEPRINT healthy B cells. Each line of *lower* panels represents the ChIP-seq chromatin states for myeloma cell lines KMS11 and MM1S and seven MM patients (P1–P7; patient-derived xenograft material) for *CCND1* (*A*) and *MAF* (*B*) loci. Numbers in colored squares (red denotes high expression; blue, low expression) show gene expression detected by RNA-seq and displayed as Log_2_-normalized counts (Log_2_ NC). (GC) Germinal center. (*) Sample contains chromosomal translocation involving the displayed region, described in detail in Supplemental Tables S3 and S5.

To investigate whether the epigenomic translocation of the *IGH* H3K4me3-BD can occur over additional proto-oncogenes in B cell malignancies, we generated ChIP-seq data for the same six histone marks for an additional two MM cell lines (KMS11 and MM1S) and seven MM patients whose diagnostic samples were engrafted in murine models (patient-derived xenografts [PDXs]). Each cell line had a complex rearrangement identified by targeted sequencing of the *IGH* locus involving multiple proto-oncogenes (Supplemental Table S3). 10x Genome sequencing detected in patients P1 and P2 the second most common *IGH* translocation in MM, t(14;16)(q32;q23), involving the transcription factor *MAF*. Patients P3 and P4 had *IGH* translocations t(4;14)(p16;q32), involving *FGFR3* and *NSD2*, with the remaining three patients (P5–P7) having no detectable translocation involving any of the Ig loci (Supplemental Table S5). No cell lines or patients had an *IGH-CCND1* rearrangement detected by targeted or genome sequencing; therefore, as expected, we did not observe an aberrant H3K4me3-BD or an increase in mRNA transcript levels for *CCND1* in these samples (Fig. 4A). This suggests that epigenomic translocations of H3K4me3-BDs over *CCND1* are a direct result of a specific genomic translocation event.

In the two cell lines and four of the patient samples (P1–P4), we observed an H3K4me3-BD over the coding region of *MAF* that was coupled with higher *MAF* expression observed by RNA-seq (Fig. 4B). This was associated with the presence of the *IGH-MAF* rearrangement in the two cell lines and patients P1 and P2. Our targeted sequencing identified the involvement of *MYC* as well in the complex translocations observed in KMS11 and MM1S cell lines (Supplemental Table S3). We observed a Polycomb chromatin state over *MYC*, but the analysis of each histone modification separately identified an H3K4me3-BD over *MYC* in the two cell lines (Supplemental Figs. S7A, S8A), possibly reflecting repression of one allele of *MYC* and activation of the other owing to being juxtaposed to an *IGH* super-enhancer (Affer et al. 2014).

In the remaining two patients (P3, P4), we were unable to detect a genomic rearrangement of a super-enhancer in close proximity to *MAF*; however, we see overexpression of *MAF* in association with an H3K4me3-BD (Fig. 4B). These two patients have the translocation t(4;14)(p16;q32) that involves *FGFR3* and *NSD2* genes. We observed an H3K4me3-BD and increased transcript levels for *FGFR3* and *NSD2*, and a Polycomb chromatin state without H3K4me3-BD was observed over *MYC* coupled with low *MYC* expression in both patients (Supplemental Figs. S7B, S8B).

In summary, we have observed the appearance of H3K4me3-BDs over a variety of proto-oncogenes when they are involved in the hijacking of an *IGH* super-enhancer. Although the chromatin state model (Carrillo-de-Santa-Pau et al. 2017) identified BDs over *CCND1* and *MAF*, it preferentially selected a Polycomb chromatin state for both *MYC* and *FGFR3* in some samples. The presence of both H3K27me3 and H3K4me3 domains over these genes potentially highlights the repression of the second allele (Nag et al. 2015), a mechanism the cell can use to control the level of overexpression. With the H3K4me3-BD signature observed over *MYC* in both healthy and malignant B cells in this study and that of Belhocine and coworkers (2022), future work will be required to unravel the epigenetic impact upon this locus after different super-enhancer translocations.

### Super-enhancer-driven H3K4me3-BDs as a wider phenomenon associated with oncogene deregulation in hematological malignancies

**Figure 5.**
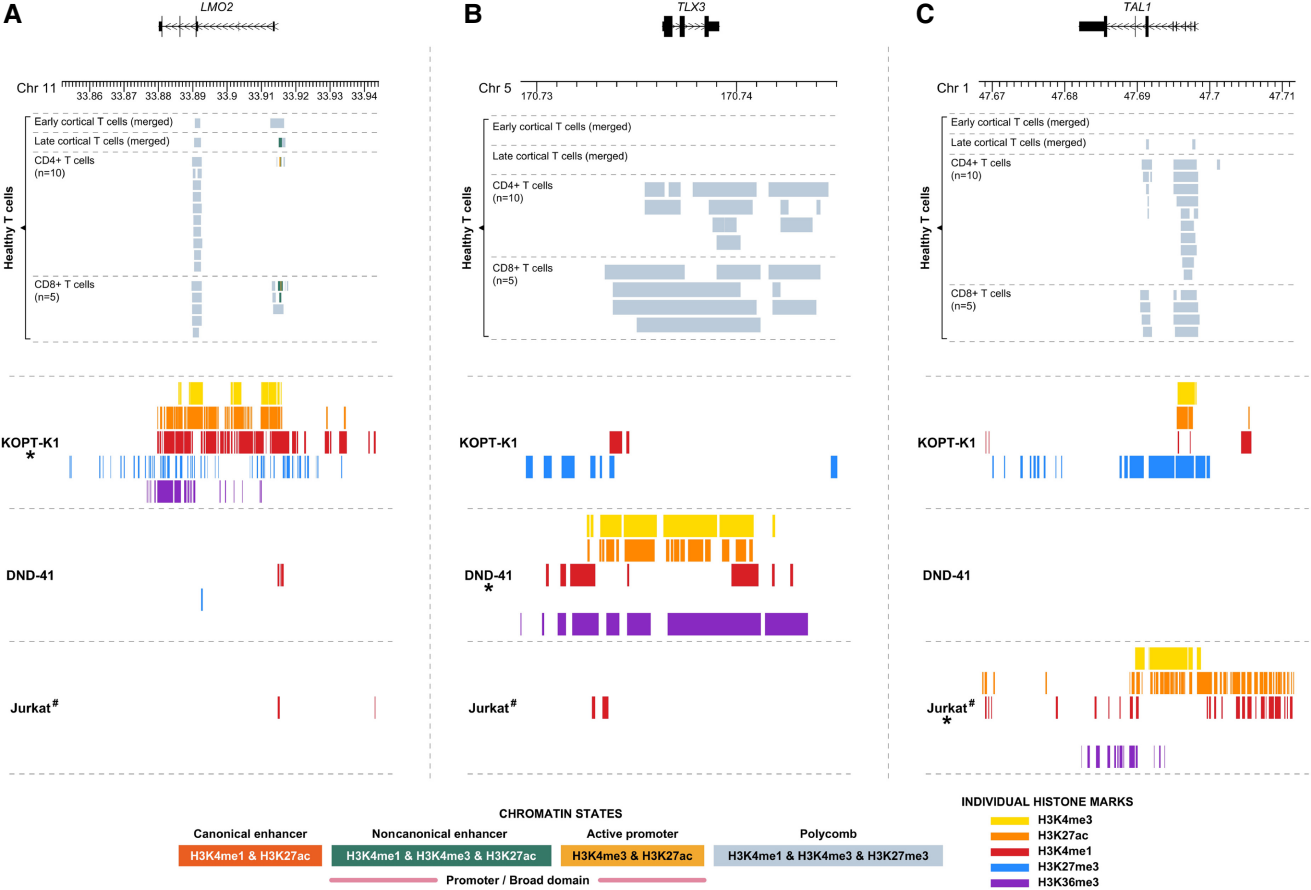
Chromatin landscape of the *LMO2* (11p13), *TLX3* (5q35), and *TAL1* (1p33) loci in healthy mature T cells and three cell lines derived from T cell hematological malignancies. *Upper* panels show selected ChIP-seq chromatin states (Supplemental Table S1) of *LMO2* (*A*), *TLX3* (*B*), and *TAL1* (*C*) loci in BLUEPRINT healthy mature T cells. Each line of the *lower* panels represents peaks of individual histone marks, separately for each cell line. (*) Cell line contains chromosomal aberration involving the displayed region as follows: KOPT-K1, *LMO2*-*TRA*/*TRD*; DND-41, *TLX3*-*BCL11B*; and Jurkat, 12-bp insertion upstream of the *TAL1* gene (for details, see Supplemental Table S3). (#) Missing data for histone mark H3K27me3 in the Jurkat cell line.

T cell receptor loci (*TRA, TRD, TRB, TRG*) are also regulated by super-enhancers, undergo similar inherent somatic rearrangement events, and are involved in translocation events in T cell acute lymphoblastic leukemia (T-ALL) (Supplemental Fig. S9; Larmonie et al. 2013). To investigate whether these loci also have the ability to generate H3K4me3-BDs over proto-oncogenes involved in translocation events, we studied the KOPT-K1 T-ALL cell line that results in the juxtaposition of *LMO2* and the super-enhancer of the *TRA/TRD* locus as a result of the translocation, t(11;14)(q13;q11). The Polycomb chromatin state was observed in healthy early and late cortical T cells and *CD4* and *CD8A* expressing T cells (Fig. 5A). We indeed observed an aberrant H3K4me3-BD over the coding region of *LMO2* in KOPT-K1, suggesting that T cell receptor super-enhancers can also generate H3K4me3-BD that results in proto-oncogene activation and overexpression (Fig. 5A; Sanda et al. 2012). An H3K4me3-BD was not observed over *LMO2* in two additional cell lines that do not have rearrangements involving this locus.

To investigate the wider involvement of H3K4m3-BDs in T-ALL, we considered the T cell identity (Ha et al. 2017) and tumor suppressor gene *BCL11B*, which is rearranged in T-ALL (Liu et al. 2017). Because this gene is known to be regulated by a downstream super-enhancer element (Supplemental Figs. S10, S11A; Nagel et al. 2007; Li et al. 2013), we questioned whether the relocation of this region from the Chromosome 14 band q32 next to *TLX3* on Chromosome 5 band q35 would result in the appearance of an aberrant H3K4me3-BD over the coding region of the gene. Using publicly available ChIP-seq data for T-ALL cell line DND-41 (Knoechel et al. 2014), we observed high signal for H3K27ac, H3K4me3, and H3K4me1, encompassing the entire coding region of *TLX3* and noncoding regions on either side (Fig. 5B). This correlated with a high transcript level compared with healthy cells (Petryszak et al. 2016). We did not observe the presence of these histone marks in normal healthy T cells and cell lines without rearrangements of these loci (Fig. 5B).

Next, we wanted to assess whether de novo super-enhancers, generated by somatic nucleotide insertions, could also generate aberrant H3K4me3-BD. An aberrant H3K4me3-BD was observed across the *TAL1* gene in the T-ALL cell line Jurkat (Fig. 5C), where a 12-bp insertion generates a super-enhancer element upstream in the gene (Mansour et al. 2014; Navarro et al. 2015). No super-enhancer and/or H3K4me-BD activity was observed upstream of the *TAL1* gene in healthy T cells (Supplemental Fig. S12). To provide mechanistic data to support the generation of H3K4me3-BD by super-enhancer relocation, we used our previously CRISPR-Cas9 engineered T-ALL cell line PEER, which contains the same 12-bp insertion upstream of the *TAL1* gene, resulting in *TAL1* overexpression (Navarro et al. 2015). H3K4me3 ChIP-seq confirmed broader H3K4 methylation upstream and over the gene body of *TAL1* in the engineered cells compared with wild type (Supplemental Fig. S13).

In summary, we found that the appearance of H3K4me3-BDs is associated with cancer-specific super-enhancer activation in both B cell– and T cell–derived hematological malignancies. Our data suggest that H3K4me3-BDs could be a signature of super-enhancer targets in general and a useful marker to identify deregulated genes affected by the hijacking of super-enhancers in cancer.

## Discussion

We aimed to understand how the juxtaposition of super-enhancers and proto-oncogenes results in exceptionally high, but restricted levels of oncogene expression. For this, we first dissected the epigenomic landscape of two loci that are commonly translocated in hematological malignancies: *CCND1* at 11q13 and the *IGH* locus at 14q32. We have shown that genomic relocation of the *IGH* Eɑ1 super-enhancer alters the location of an H3K4me3-BD in rearranged malignant B cells. The presence of broad H3K27ac domains over *CCND1* have previously been associated with t(11;14) translocations in MM cell lines including U266 (Jin et al. 2018), but this study did not use H3K4me3 data. These domains were described as de novo (genic) super-enhancers associated with *CCND1* overexpression, but they are likely showing the H3K4me3-BDs we describe here (defined as chromatin states with high levels of H3K4me3 and H3K27ac). We also observed H3K4me3-BDs over additional proto-oncogenes rearranged in B cell and T cell malignancies, including the involvement of gene-specific and de novo super-enhancers. H3K4me3-BDs are associated with high levels of stable transcription of cell-identity and tumor suppressor genes (Pekowska et al. 2010, 2011; Benayoun et al. 2014; Chen et al. 2015), and we show here, for the first time, that they can be a feature of oncogene activation by translocated super-enhancers (as a result of a genomic rearrangement).

We analyzed a total of 14 hemato-oncology samples, newly generated data for seven PDX samples, and seven patient-derived cell lines. Twelve of these cases were positive for a genomic abnormality involving hijacking of super-enhancers and proto-oncogene activation (the remaining two did not have these rearrangements). All 12 samples showed an H3K4me3-BD over the oncogene specific to a genomic rearrangement, confirming our hypothesis about the association of H3K4me3-BD and super-enhancers. In three of these 12 samples, additional H3K4me3-BDs were observed over other oncogenes not involved in a genomic rearrangement; however, there was no evidence of super-enhancer translocations at these loci. This could be because of unidentified chromosomal abnormalities involving other super-enhancers or by the existence of additional mechanisms associated with H3K4me3-BD formation. This is in agreement with our complementary publication by Belhocine and coworkers (2022), in which we observed the generation of H3K4me3-BD over many cancer-related genes, suggesting the presence of additional mechanisms able to lay down H3K4me3-BDs over coding and noncoding regions of the genome. With super-enhancers hijacking essential transcription factors (*MYC*) and chromatin remodelers (*NSD2*), it may not be surprising that the increased expression and presence of the translated protein could indirectly drive the appearance of H3K4me3-BD over other cancer-associated genes.

**Figure 6.**
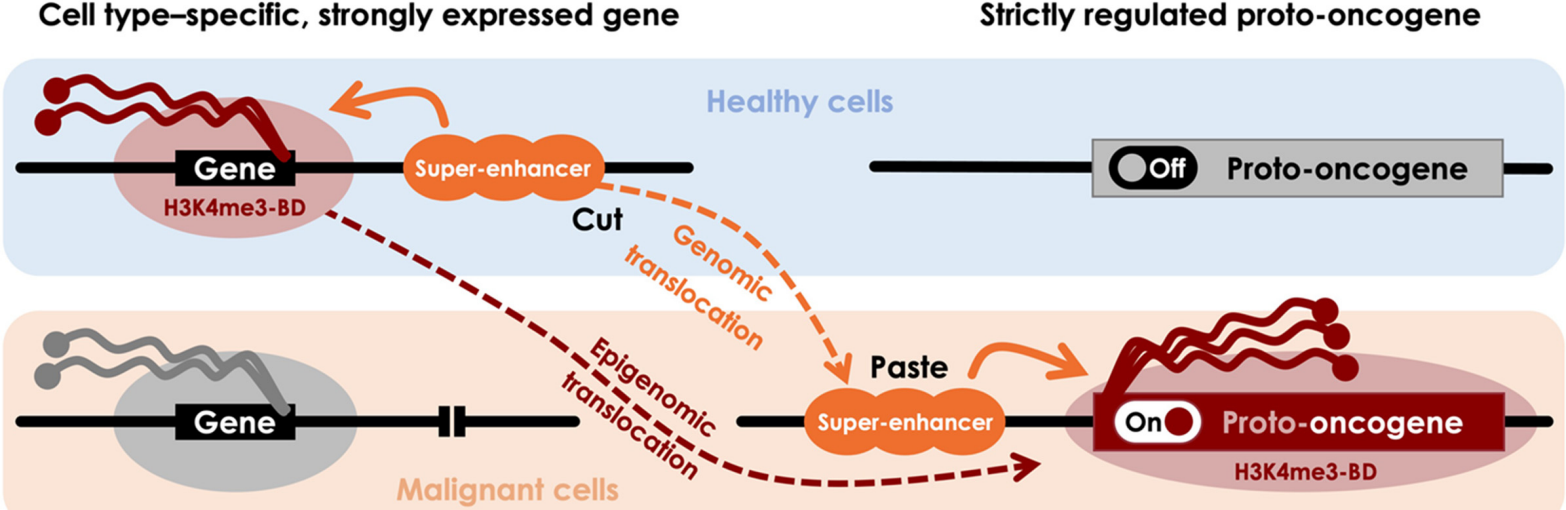
Scheme of epigenomic translocation model. In healthy cells, a cell-identity or tumor suppressor gene is expressed by regulation of a super-enhancer that generates an H3K4me3 broad domain (H3K4me3-BD), allowing rapid and consistent activation of the transcriptional machinery (*upper left*). Proto-oncogenes are strictly regulated to control important physiological processes including the cell cycle (*upper right*). During a genomic translocation event, a super-enhancer is juxtaposed close to a proto-oncogene, resulting in oncogenic activation (*bottom right*). Relocation of the super-enhancer brings the transcriptional machinery, including the H3K4me3-BD. This epigenomic signature disappears at the original locus (*bottom left*).

Based on these data, we present a model of *epigenomic translocation*, a consequence of hijacking of super-enhancers. In this model, a super-enhancer-driven wild-type H3K4me3-BD “relocates” to a target proto-oncogene as the result of a genomic rearrangement, leading to oncogene activation (Fig. 6).

Our model implies that H3K4me3-BDs can be generated by super-enhancers in their wild-type neighborhood. The H3K4me3 writer MLL4 is essential for the maintenance of both H3K4me3-BDs and super-enhancers but not for narrow-peak promoters and standard enhancers (Dhar et al. 2018). We have recently shown that inhibition of transcriptional elongation machinery by targeting CDK7 and DOT1L, or by inhibiting H3K4me3 demethylation with the use of PBIT inhibitors, can preferentially impact H3K4me4-BD and cellular viability (Belhocine et al. 2022). Initially, a strong emphasis was placed on the distinction between H3K4me3-BDs and super-enhancers (Benayoun et al. 2014) even if they were later shown to be close or sometimes overlapping in the linear genome (Chen et al. 2015; Cao et al. 2017) and in 3D (Thibodeau et al. 2017). Although super-enhancers may be responsible for generating broad domains, very few studies have addressed the length and maintenance of these regions.

Recent simulations and experimental data suggest that very active enhancers may induce high levels of gene expression by global chromatin decompaction that may actually increase the distance between promoter and enhancer regions (Buckle et al. 2018). We have observed that the presence of the H3K4me3-BD over *CCND1* coincides with high chromatin accessibility of the whole gene body. If super-enhancers induce unusual chromatin decompaction and access to DNA, this could explain how the target genes of oncogenic translocations can show such high levels of gene expression. The effects of the super-enhancer could be restricted within the boundaries of the topologically associated domains (TADs), where the hijacked oncogene and super-enhancer reside (Gong et al. 2018). Indeed, it has been suggested that one reason why TAD boundaries are conserved across species (Krefting et al. 2018; Lazar et al. 2018) could be that they prevent undesirable enhancer hijacking (Fudenberg and Pollard 2019). In related work, we have used the highly-predictive heteromorphic polymer (HiP-HoP) model (Buckle et al. 2018) to predict chromatin conformations at the proto-oncogene *CCND1* in healthy and malignant B cells (Rico et al. 2022). Our simulations of U266 and Z-138 cancer cell lines harboring *IGH-CCND1* rearrangements predict extensive changes in enhancer–promoter interactions, providing additional evidence that the downstream chromatin remodeling is essential for oncogene overexpression. In U266 cells, the TAD structure was unchanged, with the *IGH* super-enhancer providing strong predicted interactions with the promoter and gene body of *CCND1* (Rico et al. 2022). In Z-138 cells, the reciprocal translocation generated a new oncogenic TAD on Chromosome 14, with the strongest interactions observed between the *IGH* Eμ and Eδ super-enhancers, again with the promoter and gene body of *CCND1* (Rico et al. 2022). However, it is important to note that the H3K4me3-BD is not necessarily causing the oncogene overexpression, and it could actually be a consequence of the super-enhancer-driven overexpression of the oncogene (Howe et al. 2017).

The epigenomic translocation scenario is a simple yet powerful conceptual model that extends the original enhancer adoption/hijacking model (Lettice et al. 2011; Northcott et al. 2014; Beroukhim et al. 2016; Weischenfeldt et al. 2017) and allows inference of candidate genes deregulated as result of translocation events. Although we propose that some broad H3K4me3 domains are the consequence of active super-enhancers, future studies will be needed to elucidate the possible cooperation between genetic features, other epigenetic marks, the broad domain, and the super-enhancer chromatin structures and how they maintain each other.

This work will hopefully pave the way for new therapeutic approaches based on chromatin remodeling to revert the local epigenome of super-enhancer activated proto-oncogenes, returning their expression back to wild-type levels. We anticipate the model proposed here will focus attention on the regulatory effects of different genomic rearrangements including translocations, identifying key oncogenes in each patient and open exciting new avenues for novel diagnostic and therapeutic approaches.

## Methods

### ChIP-seq, DNase I hypersensitivity, and RNA-seq of BLUEPRINT data set

Previously processed ChIP-seq chromatin state BLUEPRINT samples (n = 108) were used (Supplemental Tables S2, S3). This data set was composed of 85 samples from healthy donors (three hematopoietic stem cells, 15 B cell lineage, 17 T cell lineage, and 50 myeloid lineage) and 23 samples from patients (16 primary and seven cell lines) with different B cell hematological malignancies. Five of these cell lines had DNase I hypersensitivity and RNA-seq data available (Supplemental Table S3). BLUEPRINT data were downloaded from ftp://ftp.ebi.ac.uk/pub/databases/blueprint/ paper_data_sets/chromatin_states_carrillo_build37 (ChIP-seq chromatin states) and ftp://ftp.ebi.ac.uk/pub/databases/blueprint/data/homo_sapiens/GRCh37 (DNase I hypersensitivity and RNA-seq). Early and late cortical T cell ChIP-seq chromatin states are accessible from GitHub (https://github.com/guillaumecharbonnier/mw-cieslak2019/tree/master/src/hub). Detailed laboratory methods and data processing of BLUEPRINT experiments are described elsewhere (Fernández et al. 2016; Stunnenberg et al. 2016; Carrillo-de-Santa-Pau et al. 2017; Cieslak et al. 2020). Briefly, sequencing reads were mapped using BWA (Li 2013) (ChIP-seq and DNase-seq, v0.5.9) or GEM mapper (RNA-seq) (Marco-Sola et al. 2012) to GRCh37 human genome assembly. ChromHMM (v1.10) (Ernst and Kellis 2012) was used to determine chromatin states, based on a combination of six histone modifications as follows: H3K4me1, H3K4me3, H3K9me3, H3K27me3, H3K27ac, and H3K36me3 (Carrillo-de-Santa-Pau et al. 2017). Selected chromatin states used in this study are described in Supplemental Table S1. The presence of the *IGH-CCND1* rearrangement in MCL samples was detected by conventional cytogenetics and fluorescence in situ hybridization at the Laboratory of Oncohematological Cytogenetics of Hospital Clinic Barcelona, Spain.

### Epigenomic consensus determined by BLUEPRINT ChIP-seq data

Epigenomic annotation of *IGH* (super-enhancers, promoters, and H3K4me3-BD) and *CCND1* (promoter and Polycomb) loci was built using 15 BLUEPRINT B cell/plasma-cell samples derived from healthy donors (Supplemental Table S2). Epigenomic consensus of super-enhancers and H3K4me3-BDs at *TRA/TRD* and *BCL11B* loci was assembled using 17 BLUEPRINT T cell samples derived from healthy donors. MCL (JVM-2 and Z-138) and MM (U266) cell lines with known *IGH-CCND1* rearrangements (Supplemental Table S3) were used to determine the relocated *CCND1* H3K4me3-BD.

Segmentation files of relevant samples were filtered for the subset of chromatin states (Carrillo-de-Santa-Pau et al. 2017) of interest as follows: active canonical enhancer (state 9) for super-enhancers, repressed Polycomb regulatory region (state 7) for Polycomb, and a combination of active promoter (state 11) and active noncanonical H3K4me3 enhancer (state 10) for promoters and H3K4me3-BDs. For each of these epigenomic elements, subsets were intersected and merged (both BEDTools v2.27.1) (Quinlan and Hall 2010) into continuous regions with skipping gaps <1 kb. Super-enhancers were called as regions >2.5 kb at *IGH* (overlapping the C_H_ and *IGH* intronic region), *TRA/TRD* (constant and joining regions), and *BCL11B* (from the gene position up to ∼1 Mb downstream) loci. Promoters were determined as a segment >2.5 kb within the expected promoter positions of *IGH* (C_H_ and intronic region) and *CCND1* genes. H3K4me3-BDs were scanned at *IGH* C_H_, J_H_ and D_H_, and *CCND1*, *TRA/TRD*, and *BCL11B* loci, with a minimum size of 15 kb. Polycomb at the *CCND1* locus was obtained as regions upstream and overlapping with the promoter.

### Genome-wide H3K4me3-BD and super-enhancer co-occurrence

We used an epigenomic consensus approach to test whether H3K4me3-BDs co-occur with super-enhancers at the genome-wide level in the BLUEPRINT data set (Supplemental Tables S2, S3). For this analysis, the proximity of H3K4me3-BDs (≥2 kb) to super-enhancers was compared (≥5 kb) to a control group of small promoters (1 kb; overlapping the transcription start site). Super-enhancers overlapping directly H3K4me3-BD and/or genes with H3K4me3-BD were excluded from the analysis. Also, promoters in genes with H3K4me3-BD in alternative transcription start sites were excluded. Genomic distances between H3K4me3-BD/promoters and super-enhancers up to 5 Mb were analyzed by 100-kb bins. Gene enrichment for biological processes was performed using R package clusterProfiler v3.18.0 (Yu et al. 2012). To determine epigenomic cell type–specificity, H3K4me3-BDs were tested for overlaps, and coverage of an active chromatin state background for each H3K4me3-BD was calculated. A threshold <10% was used for low active chromatin state background coverage.

### Targeted DNA sequencing of myeloma cell lines

DNA of myeloma cell lines U266, KMS11, and MM1S (Supplemental Table S3) were sequenced using targeted high-throughput sequencing covering the following genomic regions: (1) 4.2 Mb, extended coverage for chromosomal abnormality detection within *IGH*, *IGK*, *IGL*, and *MYC* (4.2 Mb); and (2) 0.6 Mb, exonic regions of 127 myeloma-specific genes for mutation analysis and 27 additional regions for efficient data normalization. GRCh37 human genome assembly was used for sequence mapping using BWA-MEM (v0.7.12). Chromosomal rearrangements were called using Manta v.0.29.6 (Chen et al. 2016; Mikulasova et al. 2020). Detailed methods were described previously (Mikulasova et al. 2020).

### Myeloma PDXs

PDXs were generated by passaging primary patient CD138 + selected cells through the previously described SCID-rab myeloma mouse model (Yata and Yaccoby 2004; Mikulasova et al. 2020). Detailed methods were described previously (Mikulasova et al. 2020). Seven PDXs were used in this study (Supplemental Table S5).

### Whole-genome sequencing of PDX samples

DNA from seven PDX samples were sequenced using phased whole-genome sequencing (10x Genomics) at Hudson Alpha. Long ranger (10x Genomics) pipelines were used for data processing, including alignment to GRCh38 genome assembly and structural variant calling. Germline controls were used to distinguish somatic abnormalities, and chromosomal breakpoints of detected translocations (Supplemental Table S5) were manually inspected.

### ChIP-seq of myeloma cell lines and PDX samples

ChIP-seq was performed as previously reported (Mikulasova et al. 2020), for the myeloma cell lines KMS11 and MM1S, as well as seven myeloma PDX samples. ChIP-seq for the histone marks H3K4me1, H3K4me3, H3K9me3, H3K27me3, H3K27ac, and H3K36me3 (Active Motif) were included in this study. Controls without antibody input were performed to ensure data quality. GRCh38 human assembly was used for alignment.

ChIP-seq chromatin states were determined by ChromHMM (v1.20), the model used in the BLUEPRINT data set (Carrillo-de-Santa-Pau et al. 2017). Additionally, the peaks of individual histone marks were detected using MACS2 (v2.2.5) (Zhang et al. 2008) by a pipeline available at the BLUEPRINT data coordination center portal (Fernández et al. 2016). For direct comparison of this data and BLUEPRINT data, the liftOver (University of California, Santa Cruz) tool was used to convert chromatin state segments and histone peaks within the genomic regions included in this study from GRCh38 to GRCh37 genome assembly. No segment was lost during this conversion.

### RNA-seq of PDX samples

RNA-seq was performed using 100 ng total RNA with genomic DNA removal using the TURBO DNA-free kit (Ambion). RNA was prepared using the TruSeq stranded total RNA Ribo-Zero gold kit (Illumina), and libraries were sequenced using 75-bp paired-end reads on a NextSeq 500 (Illumina). RNA-seq data were analyzed as previously reported (Mikulasova et al. 2020). Briefly, raw data were aligned to the human genome assembly GRCh38 with gene transcript quantification being processed by STAR (Dobin et al. 2013) (v2.5.1b) and Salmon (v0.6.0) algorithms (Patro et al. 2017). Read counts per gene were read into R and using the DESeq2 (v1.20.0) R library, normalized across samples, and the log_2_ expression calculated.

### ChIP-seq of T-ALL cell lines

Publicly available ChIP-seq data, accessed under the NCBI Gene Expression Omnibus (GEO; https://www.ncbi.nlm.nih.gov/geo/) accession numbers GSE54379 and GSE65687, were used for histone modification analysis of three T-ALL cell lines KOPT-K1, DND-41, and Jurkat (Supplemental Table S3). Raw files were downloaded using the associated NCBI Sequence Read Archive (SRA; https://www.ncbi.nlm.nih.gov/sra) accession and converted to FASTQ files using NCBI fasterq-dump (SRA Toolkit v2.9.6-1). Appropriate sequencing runs were merged, and sequences were mapped to the GRCh37 genome assembly using BWA (v0.7.17), followed by processing to MACS2 (v2.2.5) (Zhang et al. 2008) peaks, using a pipeline available at the BLUEPRINT data coordination center portal (http://dcc.blueprint-epigenome.eu/). Histone marks H3K4me1, H3K4me3, H3K27me3, H3K27ac, and H3K36me3 in all three cell lines were used in this study, with the exception of H3K27me3 in Jurkat as this was not available.

Additionally, wild type and CRISPR-Cas9 edited (upstream 12-bp insertion known from Jurkat cell line) PEER cell lines were included. The genome editing method was published elsewhere (Navarro et al. 2015). ChIP-seq for H3K4me3 and H3K27ac in PEER cell lines was generated using the MicroPlex library preparation kit (Diagenode) according to the BLUEPRINT protocol (http://dcc.blueprint-epigenome.eu/#/md/methods). Prepared libraries were sequenced in paired-end 50 + 30 bp mode using the NextSeq 500/550 (Illumina) according to the manufacturer's instructions. FASTQ files were processed by BLUEPRINT pipeline as T-ALL cell lines above. Raw read counts were collected using the Genomic Analysis Toolkit v4.2 (McKenna et al. 2010) in 200-bp bins and normalized by average number of reads per a bin.

### Statistical analysis and graphical output

Statistical analysis was performed using R v4.0.3 (R Core Team 2020). *P*-values < 0.05 were considered statistically significant. Figures involving data and annotation alignment to the human genome were generated using karyoploteR (v1.16.0) R package (Gel and Serra 2017).

### Human genome assembly statement

This study focuses on human genome loci for which differences between the hg19/GRCh37 and hg38/GRCh38 genome assemblies would not affect the results and conclusions.

## Data access

The DNA sequencing data for the U266, KMS11, and MM1S myeloma cell lines generated in this study have been submitted to the NCBI BioProject database (https://www.ncbi.nlm.nih.gov/bioproject/) under accession number PRJNA635269. All raw and processed ChIP-seq data for the KMS11 and MM1S myeloma cell lines, as well as the PEER T-ALL cell line, have been submitted to the NCBI Gene Expression Omnibus (GEO; https://www.ncbi.nlm.nih.gov/geo/) under accession number GSE151556. All high-throughput sequencing data for the MM PDXs have been submitted to the European Genome-phenome Archive (https://ega-archive.org/) under accession number EGAS00001005684.

## Supplementary Material

Supplemental Material

## References

[GR276042MIKC1] Affer M, Chesi M, Chen W-DG, Keats JJ, Demchenko YN, Roschke AV, Van Wier S, Fonseca R, Leif Bergsagel P, Michael Kuehl W. 2014. Promiscuous MYC locus rearrangements hijack enhancers but mostly super-enhancers to dysregulate MYC expression in multiple myeloma. Leukemia 28: 1725–1735. 10.1038/leu.2014.70 24518206 PMC4126852

[GR276042MIKC2] Avet-Loiseau H, Attal M, Moreau P, Charbonnel C, Garban F, Hulin C, Leyvraz S, Michallet M, Yakoub-Agha I, Garderet L, 2007. Genetic abnormalities and survival in multiple myeloma: the experience of the Intergroupe Francophone du Myélome. Blood 109: 3489–3495. 10.1182/blood-2006-08-040410 17209057

[GR276042MIKC3] Belhocine M, Simonin M, Abad Flores JD, Cieslak A, Manosalva I, Pradel L, Smith C, Mathieu EL, Charbonnier G, Martens JHA, 2022. Dynamics of broad H3K4me3 domains uncover an epigenetic switch between cell identity and cancer-related genes. Genome Res (this issue) **32:** 1328–1342. 10.1101/gr.266924.120 PMC9341507 34162697

[GR276042MIKC4] Benayoun BA, Pollina EA, Ucar D, Mahmoudi S, Karra K, Wong ED, Devarajan K, Daugherty AC, Kundaje AB, Mancini E, 2014. H3K4me3 breadth is linked to cell identity and transcriptional consistency. Cell 163: 1281–1286. 10.1016/j.cell.2015.10.051 28930648

[GR276042MIKC5] Beroukhim R, Zhang X, Meyerson M. 2016. Copy number alterations unmasked as enhancer hijackers. Nat Genet 49: 5–6. 10.1038/ng.3754 28029156

[GR276042MIKC6] Buckle A, Brackley CA, Boyle S, Marenduzzo D, Gilbert N. 2018. Polymer simulations of heteromorphic chromatin predict the 3D folding of complex genomic loci. Mol Cell 72: 786–797.e11. 10.1016/j.molcel.2018.09.016 30344096 PMC6242782

[GR276042MIKC7] Cao F, Fang Y, Tan HK, Goh Y, Choy JYH, Koh BTH, Tan JH, Bertin N, Ramadass A, Hunter E, 2017. Super-enhancers and broad H3K4me3 domains form complex gene regulatory circuits involving chromatin interactions. Sci Rep 7: 2186. 10.1038/s41598-017-02257-3 28526829 PMC5438348

[GR276042MIKC8] Carrillo-de-Santa-Pau E, Juan D, Pancaldi V, Were F, Martin-Subero I, Rico D, Valencia A, on behalf of The BLUEPRINT Consortium. 2017. Automatic identification of informative regions with epigenomic changes associated to hematopoiesis. Nucleic Acids Res 45: 9244–9259. 10.1093/nar/gkx618 28934481 PMC5716146

[GR276042MIKC9] Chen C, Birshtein BK. 1997. Virtually identical enhancers containing a segment of homology to murine 3′IgH-E(hs1,2) lie downstream of human Ig C*α*1 and C*α*2 genes. J Immunol 159: 1310–1318. 9233627

[GR276042MIKC10] Chen K, Chen Z, Wu D, Zhang L, Lin X, Su J, Rodriguez B, Xi Y, Xia Z, Chen X, 2015. Broad H3K4me3 is associated with increased transcription elongation and enhancer activity at tumor-suppressor genes. Nat Genet 47: 1149–1157. 10.1038/ng.3385 26301496 PMC4780747

[GR276042MIKC11] Chen X, Schulz-Trieglaff O, Shaw R, Barnes B, Schlesinger F, Källberg M, Cox AJ, Kruglyak S, Saunders CT. 2016. Manta: rapid detection of structural variants and indels for germline and cancer sequencing applications. Bioinformatics 32: 1220–1222. 10.1093/bioinformatics/btv710 26647377

[GR276042MIKC12] Cieslak A, Charbonnier G, Tesio M, Mathieu E-L, Belhocine M, Touzart A, Smith C, Hypolite G, Andrieu GP, Martens JHA, 2020. Blueprint of human thymopoiesis reveals molecular mechanisms of stage-specific TCR enhancer activation. J Exp Med 217: e20192360. 10.1084/jem.20192360 32667968 PMC7478722

[GR276042MIKC13] Dhar SS, Zhao D, Lin T, Gu B, Pal K, Wu SJ, Alam H, Lv J, Yun K, Gopalakrishnan V, 2018. MLL4 is required to maintain broad H3K4me3 peaks and super-enhancers at tumor suppressor genes. Mol Cell 70: 825–841. 10.1016/j.molcel.2018.04.028 29861161 PMC6528658

[GR276042MIKC02] Dobin A, Davis CA, Schlesinger F, Drenkow J, Zaleski C, Jha S, Batut P, Chaisson M, Gingeras TR. 2013. STAR: ultrafast universal RNA-seq aligner. Bioinformatics 29: 15–21. 10.1093/bioinformatics/bts635 23104886 PMC3530905

[GR276042MIKC14] Ernst J, Kellis M. 2012. ChromHMM: automating chromatin-state discovery and characterization. Nat Methods 9: 215–216. 10.1038/nmeth.1906 22373907 PMC3577932

[GR276042MIKC15] Fernández JM, de la Torre V, Richardson D, Royo R, Puiggròs M, Moncunill V, Fragkogianni S, Clarke L, Flicek P, Rico D, 2016. The BLUEPRINT data analysis portal. Cell Syst 3: 491–495. 10.1016/j.cels.2016.10.021 27863955 PMC5919098

[GR276042MIKC16] Fudenberg G, Pollard KS. 2019. Chromatin features constrain structural variation across evolutionary timescales. Proc Natl Acad Sci 116: 2175–2180. 10.1073/pnas.1808631116 30659153 PMC6369792

[GR276042MIKC17] Gabrea A, Leif Bergsagel P, Chesi M, Shou Y, Michael Kuehl W. 1999. Insertion of excised IgH switch sequences causes overexpression of cyclin D1 in a myeloma tumor cell. Mol Cell 3: 119–123. 10.1016/s1097-2765(00)80180-x 10024885

[GR276042MIKC04] Gel B, Serra E. 2017. karyoploteR: an R/Bioconductor package to plot customizable genomes displaying arbitrary data. Bioinformatics 33: 3088–3090. 10.1093/bioinformatics/btx346 28575171 PMC5870550

[GR276042MIKC18] Gong Y, Lazaris C, Sakellaropoulos T, Lozano A, Kambadur P, Ntziachristos P, Aifantis I, Tsirigos A. 2018. Stratification of TAD boundaries reveals preferential insulation of super-enhancers by strong boundaries. Nat Commun 9: 542. 10.1038/s41467-018-03017-1 29416042 PMC5803259

[GR276042MIKC19] Guikema JEJ, Fenton JAL, de Boer C, Kleiverda K, Brink AATP, Raap AK, Estrov Z, Schuuring E, Kluin PM. 2005. Complex biallelic IGH rearrangements in IgM-expressing Z-138 cell line: involvement of downstream immunoglobulin class switch recombination. Genes Chromosomes Cancer 42: 164–169. 10.1002/gcc.20132 15543623

[GR276042MIKC20] Ha VL, Luong A, Li F, Casero D, Malvar J, Kim YM, Bhatia R, Crooks GM, Parekh C. 2017. The T-ALL related gene *BCL11B* regulates the initial stages of human T-cell differentiation. Leukemia 31: 2503–2514. 10.1038/leu.2017.70 28232744 PMC5599326

[GR276042MIKC21] Hellman L, Josephson S, Jernberg H, Nilsson K, Pettersson U. 1988. Immunoglobulin synthesis in the human myeloma cell line U-266; expression of two immunoglobulin heavy chain isotypes (ε and α) after long-term cultivation *in vitro*. Eur J Immunol 18: 905–910. 10.1002/eji.1830180611 3133230

[GR276042MIKC22] Hnisz D, Weintraub AS, Day DS, Valton A-L, Bak RO, Li CH, Goldmann J, Lajoie BR, Fan ZP, Sigova AA, 2016. Activation of proto-oncogenes by disruption of chromosome neighborhoods. Science 351: 1454–1458. 10.1126/science.aad9024 26940867 PMC4884612

[GR276042MIKC23] Howe FS, Fischl H, Murray SC, Mellor J. 2017. Is H3K4me3 instructive for transcription activation? Bioessays 39: 1–12. 10.1002/bies.201600095 28004446

[GR276042MIKC24] Hu D, Gao X, Cao K, Morgan MA, Mas G, Smith ER, Volk AG, Bartom ET, Crispino JD, Di Croce L, 2017. Not all H3K4 methylations are created equal: Mll2/COMPASS dependency in primordial germ cell specification. Mol Cell 65: 460–475.e6. 10.1016/j.molcel.2017.01.013 28157506 PMC5404503

[GR276042MIKC25] Jin Y, Chen K, De Paepe A, Hellqvist E, Krstic AD, Metang L, Gustafsson C, Davis RE, Levy YM, Surapaneni R, 2018. Active enhancer and chromatin accessibility landscapes chart the regulatory network of primary multiple myeloma. Blood 131: 2138–2150. 10.1182/blood-2017-09-808063 29519805 PMC6014038

[GR276042MIKC26] Knoechel B, Roderick JE, Williamson KE, Zhu J, Lohr JG, Cotton MJ, Gillespie SM, Fernandez D, Ku M, Wang H, 2014. An epigenetic mechanism of resistance to targeted therapy in T cell acute lymphoblastic leukemia. Nat Genet 46: 364–370. 10.1038/ng.2913 24584072 PMC4086945

[GR276042MIKC27] Krefting J, Andrade-Navarro MA, Ibn-Salem J. 2018. Evolutionary stability of topologically associating domains is associated with conserved gene regulation. BMC Biol 16: 87. 10.1186/s12915-018-0556-x 30086749 PMC6091198

[GR276042MIKC28] Larmonie NSD, Dik WA, Meijerink JPP, Homminga I, van Dongen JJM, Langerak AW. 2013. Breakpoint sites disclose the role of the V(D)J recombination machinery in the formation of T-cell receptor (TCR) and non-TCR associated aberrations in T-cell acute lymphoblastic leukemia. Haematologica 98: 1173–1184. 10.3324/haematol.2012.082156 23904235 PMC3729897

[GR276042MIKC29] Lazar NH, Nevonen KA, O'Connell B, McCann C, O'Neill RJ, Green RE, Meyer TJ, Okhovat M, Carbone L. 2018. Epigenetic maintenance of topological domains in the highly rearranged gibbon genome. Genome Res 28: 983–997. 10.1101/gr.233874.117 29914971 PMC6028127

[GR276042MIKC30] Lettice LA, Daniels S, Sweeney E, Venkataraman S, Devenney PS, Gautier P, Morrison H, Fantes J, Hill RE, FitzPatrick DR. 2011. Enhancer-adoption as a mechanism of human developmental disease. Hum Mutat 32: 1492–1499. 10.1002/humu.21615 21948517

[GR276042MIKC01] Li H. 2013. Aligning sequence reads, clone sequences and assembly contigs with BWA-MEM. arXiv:1303.3997 [q-bio.GN].

[GR276042MIKC31] Li L, Zhang JA, Dose M, Kueh HY, Mosadeghi R, Gounari F, Rothenberg EV. 2013. A far downstream enhancer for murine *Bcl11b* controls its T-cell specific expression. Blood 122: 902–911. 10.1182/blood-2012-08-447839 23741008 PMC3739036

[GR276042MIKC32] Li Q-L, Wang D-Y, Ju L-G, Yao J, Gao C, Lei P-J, Li L-Y, Zhao X-L, Wu M. 2019. The hyper-activation of transcriptional enhancers in breast cancer. Clin Epigenetics 11: 48. 10.1186/s13148-019-0645-x 30867030 PMC6417266

[GR276042MIKC33] Liu Y, Easton J, Shao Y, Maciaszek J, Wang Z, Wilkinson MR, McCastlain K, Edmonson M, Pounds SB, Shi L, 2017. The genomic landscape of pediatric and young adult T-lineage acute lymphoblastic leukemia. Nat Genet 49: 1211–1218. 10.1038/ng.3909 28671688 PMC5535770

[GR276042MIKC34] Lovén J, Hoke HA, Lin CY, Lau A, Orlando DA, Vakoc CR, Bradner JE, Lee TI, Young RA. 2013. Selective inhibition of tumor oncogenes by disruption of super-enhancers. Cell 153: 320–334. 10.1016/j.cell.2013.03.036 23582323 PMC3760967

[GR276042MIKC35] Mansour MR, Abraham BJ, Anders L, Berezovskaya A, Gutierrez A, Durbin AD, Etchin J, Lawton L, Sallan SE, Silverman LB, 2014. Oncogene regulation: an oncogenic super-enhancer formed through somatic mutation of a noncoding intergenic element. Science 346: 1373–1377. 10.1126/science.1259037 25394790 PMC4720521

[GR276042MIKC36] Marco-Sola S, Sammeth M, Guigó R, Ribeca P. 2012. The GEM mapper: fast, accurate and versatile alignment by filtration. Nat Methods 9: 1185–1188. 10.1038/nmeth.2221 23103880

[GR276042MIKC03] McKenna A, Hanna M, Banks E, Sivachenko A, Cibulskis K, Kernytsky A, Garimella K, Altshuler D, Gabriel S, Daly M, 2010. The Genome Analysis Toolkit: a MapReduce framework for analyzing next-generation DNA sequencing data. Genome Res 20: 1297–1303. 10.1101/gr.107524.110 20644199 PMC2928508

[GR276042MIKC37] Mikulasova A, Ashby C, Tytarenko RG, Qu P, Rosenthal A, Dent JA, Ryan KR, Bauer MA, Wardell CP, Hoering A, 2020. Microhomology-mediated end joining drives complex rearrangements and over expression of *MYC* and *PVT1* in multiple myeloma. Haematologica 105: 1055–1066. 10.3324/haematol.2019.217927 31221783 PMC7109748

[GR276042MIKC38] Mills FC, Fisher LM, Kuroda R, Ford AM, Gould HJ. 1983. DNase I hypersensitive sites in the chromatin of human µ immunoglobulin heavy-chain genes. Nature 306: 809–812. 10.1038/306809a0 6419125

[GR276042MIKC39] Mills FC, Harindranath N, Mitchell M, Max EE. 1997. Enhancer complexes located downstream of both human immunoglobulin Cα genes. J Exp Med 186: 845–858. 10.1084/jem.186.6.845 9294139 PMC2199054

[GR276042MIKC40] Nag A, Vigneau S, Savova V, Zwemer LM, Gimelbrant AA. 2015. Chromatin signature identifies monoallelic gene expression across mammalian cell types. G3 (Bethesda) 5: 1713–1720. 10.1534/g3.115.018853 26092837 PMC4528328

[GR276042MIKC41] Nagel S, Scherr M, Kel A, Hornischer K, Crawford GE, Kaufmann M, Meyer C, Drexler HG, MacLeod RAF. 2007. Activation of *TLX3* and *NKX2-5* in t(5;14)(q35;q32) T-cell acute lymphoblastic leukemia by remote 3′-*BCL11B* enhancers and coregulation by PU.1 and HMGA1. Cancer Res 67: 1461–1471. 10.1158/0008-5472.CAN-06-2615 17308084

[GR276042MIKC42] Navarro J-M, Touzart A, Pradel LC, Loosveld M, Koubi M, Fenouil R, Le Noir S, Maqbool MA, Morgado E, Gregoire C, 2015. Site- and allele-specific Polycomb dysregulation in T-cell leukaemia. Nat Commun 6: 6094. 10.1038/ncomms7094 25615415 PMC4317503

[GR276042MIKC43] Northcott PA, Lee C, Zichner T, Stütz AM, Erkek S, Kawauchi D, Shih DJH, Hovestadt V, Zapatka M, Sturm D, 2014. Enhancer hijacking activates GFI1 family oncogenes in medulloblastoma. Nature 511: 428–434. 10.1038/nature13379 25043047 PMC4201514

[GR276042MIKC05] Patro R, Duggal G, Love MI, Irizarry RA, Kingsford C. 2017. Salmon provides fast and bias-aware quantification of transcript expression. Nat Methods 14: 417–419. 10.1038/nmeth.4197 28263959 PMC5600148

[GR276042MIKC44] Pekowska A, Benoukraf T, Ferrier P, Spicuglia S. 2010. A unique H3K4me2 profile marks tissue-specific gene regulation. Genome Res 20: 1493–1502. 10.1101/gr.109389.110 20841431 PMC2963813

[GR276042MIKC45] Pekowska A, Benoukraf T, Zacarias-Cabeza J, Belhocine M, Koch F, Holota H, Imbert J, Andrau J-C, Ferrier P, Spicuglia S. 2011. H3K4 tri-methylation provides an epigenetic signature of active enhancers. EMBO J 30: 4198–4210. 10.1038/emboj.2011.295 21847099 PMC3199384

[GR276042MIKC46] Petryszak R, Keays M, Amy Tang Y, Fonseca NA, Barrera E, Burdett T, Füllgrabe A, Fuentes AM-P, Jupp S, Koskinen S, 2016. Expression atlas update: an integrated database of gene and protein expression in humans, animals and plants. Nucleic Acids Res 44: D746–D752. 10.1093/nar/gkv1045 26481351 PMC4702781

[GR276042MIKC47] Pott S, Lieb JD. 2015. What are super-enhancers? Nat Genet 47: 8–12. 10.1038/ng.3167 25547603

[GR276042MIKC48] Quinlan AR, Hall IM. 2010. BEDTools: a flexible suite of utilities for comparing genomic features. Bioinformatics 26: 841–842. 10.1093/bioinformatics/btq033 20110278 PMC2832824

[GR276042MIKC49] R Core Team. 2020. R: a language and environment for statistical computing. R Foundation for Statistical Computing, Vienna. https://www.R-project.org/.

[GR276042MIKC50] Rico D, Kent D, Karataraki N, Mikulasova A, Berlinguer-Palmini R, Walker BA, Javierre BM, Russell LJ, Brackley CA. 2022. High-resolution simulations of chromatin folding at genomic rearrangements in malignant B cells provide mechanistic insights into proto-oncogene deregulation. Genome Res (this issue) 32: 1355–1366. 10.1101/gr.276028.121 PMC9341513 35863900

[GR276042MIKC51] Russ BE, Olshansky M, Li J, Nguyen MLT, Gearing LJ, Nguyen THO, Olson MR, McQuilton HA, Nüssing S, Khoury G, 2017. Regulation of H3K4me3 at transcriptional enhancers characterizes acquisition of virus-specific CD8^+^ T cell-lineage-specific function. Cell Rep 21: 3624–3636. 10.1016/j.celrep.2017.11.097 29262339

[GR276042MIKC52] Sanda T, Lawton LN, Inmaculada Barrasa M, Fan ZP, Kohlhammer H, Gutierrez A, Ma W, Tatarek J, Ahn Y, Kelliher MA, 2012. Core transcriptional regulatory circuit controlled by the TAL1 complex in human T cell acute lymphoblastic leukemia. Cancer Cell 22: 209–221. 10.1016/j.ccr.2012.06.007 22897851 PMC3422504

[GR276042MIKC53] Shen H, Xu W, Guo R, Rong B, Gu L, Wang Z, He C, Zheng L, Hu X, Hu Z, 2016. Suppression of enhancer overactivation by a RACK7-histone demethylase complex. Cell 165: 331–342. 10.1016/j.cell.2016.02.064 27058665 PMC4826479

[GR276042MIKC54] Stamatopoulos K, Kosmas C, Belessi C, Kyriazopoulos P, Papadaki T, Anagnostou D, Loukopoulos D. 1999. Molecular analysis of Bcl-1/IgH junctional sequences in mantle cell lymphoma: potential mechanism of the t(11;14) chromosomal translocation. Br J Haematol 105: 190–197. 10.1111/j.1365-2141.1999.01314.x 10233383

[GR276042MIKC55] Stunnenberg HG, International Human Epigenome Consortium, Hirst M. 2016. The International Human Epigenome Consortium: a blueprint for scientific collaboration and discovery. Cell 167: 1145–1149. 10.1016/j.cell.2016.11.007 27863232

[GR276042MIKC56] Thibodeau A, Márquez EJ, Shin D-G, Vera-Licona P, Ucar D. 2017. Chromatin interaction networks revealed unique connectivity patterns of broad H3K4me3 domains and super enhancers in 3D chromatin. Sci Rep 7: 14466. 10.1038/s41598-017-14389-7 29089515 PMC5663946

[GR276042MIKC57] Vose JM. 2017. Mantle cell lymphoma: 2017 update on diagnosis, risk-stratification, and clinical management. Am J Hematol 92: 806–813. 10.1002/ajh.24797 28699667

[GR276042MIKC58] Walker BA, Wardell CP, Johnson DC, Kaiser MF, Begum DB, Dahir NB, Ross FM, Davies FE, Gonzalez D, Morgan GJ. 2013. Characterization of *IGH* locus breakpoints in multiple myeloma indicates a subset of translocations appear to occur in pregerminal center B cells. Blood 121: 3413–3419. 10.1182/blood-2012-12-471888 23435460

[GR276042MIKC59] Weischenfeldt J, Dubash T, Drainas AP, Mardin BR, Chen Y, Stütz AM, Waszak SM, Bosco G, Halvorsen AR, Raeder B, 2017. Pan-cancer analysis of somatic copy-number alterations implicates *IRS4* and *IGF2* in enhancer hijacking. Nat Genet 49: 65–74. 10.1038/ng.3722 27869826 PMC5791882

[GR276042MIKC60] Whyte WA, Orlando DA, Hnisz D, Abraham BJ, Lin CY, Kagey MH, Rahl PB, Lee TI, Young RA. 2013. Master transcription factors and mediator establish super-enhancers at key cell identity genes. Cell 153: 307–319. 10.1016/j.cell.2013.03.035 23582322 PMC3653129

[GR276042MIKC61] Yata K, Yaccoby S. 2004. The SCID-rab model: a novel *in vivo* system for primary human myeloma demonstrating growth of CD138-expressing malignant cells. Leukemia 18: 1891–1897. 10.1038/sj.leu.2403513 15385929

[GR276042MIKC06] Yu G, Wang LG, Han Y, He QY. 2012. clusterProfiler: an R package for comparing biological themes among gene clusters. OMICS 16: 284–287. 10.1089/omi.2011.0118 22455463 PMC3339379

[GR276042MIKC07] Zhang Y, Liu T, Meyer CA, Eeckhoute J, Johnson DS, Bernstein BE, Nusbaum C, Myers RM, Brown M, Li W, 2008. Model-based Analysis of ChIP-Seq (MACS). Genome Biol 9: R137. 10.1186/gb-2008-9-9-r137 18798982 PMC2592715

