# Supplementary material for: Epigenomic translocation of H3K4me3 broad domains over oncogenes following hijacking of super-enhancers
Source: Genome Res. 2022 Jul;32(7):1343–54. doi: 10.1101/gr.276042.121 (PMC9341503; doi:10.1101/gr.276042.121)
Supplement: Supplemental Material [file supp_32_7_1343__DC1.html]

Epigenomic translocation of H3K4me3 broad domains over oncogenes following hijacking of super-enhancers — Epigenomic translocation of H3K4me3 broad domains over oncogenes following hijacking of super-enhancers — Supplemental Material 

# Epigenomic translocation of H3K4me3 broad domains over oncogenes following hijacking of super-enhancers

## Supplemental Material

- Supplemental\_Table\_S4.txt
- Supplemental\_Materials.pdf
